# Score risk model for predicting severe fever with thrombocytopenia syndrome mortality

**DOI:** 10.1186/s12879-016-2111-0

**Published:** 2017-01-07

**Authors:** Li Wang, Zhiqiang Zou, Chunguo Hou, Xiangzhong Liu, Fen Jiang, Hong Yu

**Affiliations:** Infectious Disease Hospital of Yantai, 62 Huanshan Road, Zhifu district, Yantai, Shandong 264001 China

**Keywords:** Risk model, Severe fever with thrombocytopenia syndrome, Prediction, Mortality

## Abstract

**Background:**

Severe fever with thrombocytopenia syndrome (SFTS) is an emerging epidemic infectious disease with high mortality in East Aisa, especially in China. To predict the prognosis of SFTS precisely is important in clinical practice.

**Methods:**

From May 2013 to November 2015, 233 suspected SFTS patients were tested for SFTS virus using RT-PCR. Cox regression model was utilized to comfirm independent risk factors for mortality. A risk score model for mortality was constructed based on regression coefficient of risk factors. Log-rank test was used to evaluate the significance of this model.

**Results:**

One hundred seventy-four patients were confirmed with SFTS, of which 40 patients died (23%). Baseline age, serum aspartate aminotransferase (AST) and serum creatinine (sCr) level were independent risk factors of mortality. The area under ROC curve (AUCs) of these parameters for predicting death were 0.771, 0.797 and 0.764, respectively. And hazard ratio (HR) were 1.128, 1.002 and 1.013, respectively. The cutoff value of the risk model was 10. AUC of the model for predicting mortality was 0.892, with sensitivity and specificity of 82.5 and 86.6%, respectively. Log-rank test indicated strong statistical significance (×^2^ = 88.35, *p* < 0.001).

**Conclusions:**

This risk score model may be helpful to predicting the prognosis of SFTS patients.

## Background

Severe fever with thrombocytopenia syndrome (SFTS) is an emerging epidemic infectious disease in China, Korea and Japan caused by a novel bunyavirus, SFTS virus (SFTSV). It has become an important public health threat in Asia, especially in China. The national surveillance data from 2010 to 2013 in China showed that the incidence of SFTS and its epidemic areas are growing, but the case fatality rate (CFR) has steadily declined [1]. The major clinical symptoms of SFTS include fever, thrombocytopenia, leukocytopenia, gastrointestinal symptoms and various other systemic manifestations including muscular symptoms, neurological disorders and coagulopathy [2, 3]. Neurological symptoms were strongly associated with death, even though they occurred during the first week after disease onset [4]. Other factors associated with death include viral load [5–8], advanced age, serum aspartate aminotransferase (AST), lactate dehydrogenase (LDH) and creatine kinase (CK) levels, and decreased lymphocyte percentages [7–12].

We and others [13] have found in clinical practice that though some patients had similar symptoms at admission, including altered consciousness and multiple organ dysfunctions and received similar therapy, their clinical courses were quite different. However, several patients who died afterwards did not exhibit neurological disorders at admission. Thus it is important to predict the prognosis of the patients in clinical practice.

Though the viral load is a predictor of morbidity [13], in contrast to routine laboratory examination, which is quickly and easily performed, the level of SFTS viral RNA requires several days to determine, and can only be conducted at a limited number of local disease control institutes. Furthermore, the viral RNA only becomes detectable in most cases at three days and reaches maximum level at six days after disease onset. In 55.6% of the patients, viral RNA was undetectable at hospital admission [8]. In this study, we established a risk score model for predicting mortality of SFTS patients based on their clinical and demographic characteristics, and routine laboratory tests including biochemical, hematological parameters and coagulation variables.

## Methods

### Clinical samples and laboratory testing

Patients with clinical manifestations of suspected SFTS, including fever (body temperature >38.0 °C prior to admission or at admission), thrombocytopenia, leukocytopenia and gastrointestinal symptoms were tested for SFTSV via reverse transcription–polymerase chain reaction (RT–PCR) using total RNA extracted from a peripheral blood sample [14] at local institute for disease preventation and control. The results are given as positive or negative afterwards for clinicians to confirm SFTSV infection. Clinical history and manifestation, physical examination, routine biochemical, hematological and coagulation results at enrollment were retrospectively collected from confirmed SFTS patients. Hematological parameters were detected using an XT1800I blood cell analyzer and biochemical parameters using a CI16200 blood biochemical automatic analyzer. Neurological symptoms included: limb shaking, lower jaw shaking, dysarthria and discordance of consciousness.

Parameters which entered in Cox univariate regression analysis included: age, gender, body temperature, time intervals between symptom onset to admission, hematological parameters, biochemical parameters, coagulation indicators and neurological symptom.

This study was conducted according to the Helsinki II Declaration and approved by the ethics committee at the National Institute of Parasitic Diseases, Chinese Center for Disease Control and Prevention. Written informed consent was obtained from the patients.

### Statistical analysis

Statistical analyses were performed using the SPSS, version 16.0, software (IBM, Armonk, NY, USA). Mann–Whitney *U* test was used for variables with an abnormal distribution between the survival and death groups. Cox univariate and multivariate regression analysis was applied to determine the risk factors among parameters at admission that were associated with mortality of SFTS patients. Factors with *p* < 0. 1 in univariate analysis were included in Cox multivariate regression analysis. Risk score model was established based on the acquired regression coefficients of the determined risk factors to predict mortality. Criterion for factors that include in the regression model was that the factors present in the last step of forward conditional with *p* < 0.05 and Hazard ratios (HR) >1.0. Receiver-operating characteristic (ROC) curves and area under the curve (AUC) were constructed to assess the predictive power of the model. Patients were divided into high-risk and low-risk groups according to cutoff values obtained from ROC analysis. The Kaplan-Meier survival analysis was used to compare the cumulative risk for death in the two groups, and the significance of difference was tested with the log-rank test. ×^2^ test was utilized to compare mortality rate between patients with above and less than cutoff value of risk model. *p* < 0.05 was considered to be statistically significant.

## Results

### Demographic and clinical characteristics of SFTS patients

Among the 233 suspected cases with fever, thrombocytopenia, fatigue and diarrhea admitted to our hospital from May 2013 to November 2015, 174 were finally diagnosed as SFTS by detecting SFTSV using RT-PCR. Forty patients (23%) subsequently died. The survival group included 63 women (47.0%) and 71 men (53.0%), while the non-survival group comprised of 19 women (47.5%) and 21 men (52.5%). Gender composition was identical in the two groups. The median age was 64 (range 28–84) years, and was significantly higher among those who died (range 52–84 years; *p* < 0.001).

The major baseline clinical symptoms and findings included fever (*n* = 102, 58.6%), fatigue (*n* = 120, 69.0%), loss of appetite (*n* = 86, 50%) diarrhea (*n* = 12, 6.9%), superficial lymphadenopathy (*n* = 72, 41.4%), neutropenia (*n* = 110, 63.2%), thrombocytopenia (*n* = 174, 100%) and neurological symptoms (*n* = 63, 36.2%). Among those who survived, 38 cases manifested neurological symptoms. Nine cases had severe neurological symptoms included abnormal level of consciousness. Others exhibited mainly limb shaking. Among those patients who have died, 25 cases had neurological symptoms (60%) at admission and other developed central neurological symptoms primarily discordance of consciousness and coma within 3–7 days. Ages of those with severe neurological symptoms who survived are younger than those who died (62.3 ± 7.8 vs. 70.9 ± 9.5, *p* < 0.05).

Body temperature at admission in surviving and fatal cases (37.4 ± 0.98°Cvs 37.5 ± 1.1 °C, *p* >0.05) were comparable. The time intervals between symptom onset to admission were also similar between the two groups (6.5 ± 3.3 vs 5.6 ± 1.9, *p* >0.05). Hospitalization duration was longer in patients who recovered than those who died (13.0 ± 5.5 vs 3.4 ± 2.4, *p* < 0.001).

### Comparison of biochemical and hematological parameters in surviving and fatal SFTS cases

For the biochemical parameters, levels of alanine aminotransferase (ALT), AST, total bilirubin (TB), high-sensitivity C-reactive protein (hs-CRP), blood urea nitrogen (BUN), serum creatinine (sCr), troponin (Ctnl), CK, hydroxybutyrate dehydrogenase (HBDH), LDH, prothrombin time (PT) and international normalized ratio (INR) were dramatically increased in the fatal cases. While the prothrombin time activity percentage (PTA) was markedly decreased. Concentrations of serum potassium (K^+^) and phosphorus (P^3+^) in the fatal cases were significantly elevated, whereas sodium (Na^+^), calcium (Ca^2+^) and hydrocarbonate (HCO_3_-) were notably reduced.

For the hematological parameters, white cell blood (WBC) count, neutrophil (NEU) count, neutrophil/lymphocyte (N/L) ratio, mean corpuscular volume (MCV) and red blood volume distributing width (RDW) in the fatal cases were remarkably increased. Red blood cell (RBC) count, lymphocyte (LYM) count, PLT count and thrombocytocrit (PCT) were prominently decreased. These results are shown in Tables 1 and 2.

**Table 1:** Comparison of biochemical parameters in survivals and non-survivals of STFS patients ($\overline{x}$ ±SD)

Parameters	Survival	Non-survival	*p*
N	134	40	
Male/female	71/63	21/19	>0.05
Age (year)	61.2 ± 9.9	71 ± 9.5	<0.001
Hospital stays (day)	13.0 ± 5.5	3.4 ± 2.4	<0.001
Body temperature (°C)	37.4 ± 0.98	37.5 ± 1.0	>0.05
ALT (U/L)	95.4 ± 86.7	191.8 ± 148.8	<0.001
AST (U/L)	210.3 ± 219.0	713.4 ± 572.9	<0.001
TB (μmol/L)	11.6 ± 5.0	19.0 ± 13.4	<0.001
HCO3-(mmol/L)	24.1 ± 3.0	22.6 ± 5.5	<0.05
Ca2+ (mmol/L)	1.96 ± 0.13	1.86 ± 0.17	<0.01
P3 + (mmol/L)	0.93 ± 0.25	1.04 ± 0.31	<0.05
Hs-CRP (mg/L)	9.6 ± 11.6	18.3 ± 15.7	<0.001
Dimer (ng/L)	5.2 ± 10.5	14.4 ± 22.4	<0.001
BUN (mmol/L)	4.9 ± 2.4	10.1 ± 5.9	<0.001
SCr (μmol/L)	64.1 ± 16.3	115.1 ± 79.3	<0.001
UA (μmol/L)	286.8 ± 117.6	430.4 ± 192.2	<0.001
Ctnl (ng/mL)	8.5 ± 93.6	1.2 ± 3.6	<0.001
CK (U/L)	916.7 ± 1424.4	2372.0 ± 3680.1	<0.001
CK-MB (U/L)	47.8 ± 54.8	85.5 ± 78.5	<0.001
HBDH (U/L)	4846.8 ± 326.2	967.1 ± 848.2	<0.001
LDH (U/L)	804.1 ± 811.8	1989.3 ± 1862.1	<0.001
PT (s)	12.2 ± 1.3	13.6 ± 2.0	<0.01
PTA (%)	107.1 ± 18.9	89.1 ± 17.3	<0.01
INR	0.98 ± 0.11	1.09 ± 0.17	<0.01

*ALT* alanine aminotransferase, *AST* aspartate aminotransferase, *hs-CRP* high-sensitivity C-reactive protein, *BUN* blood urea nitrogen, *sCr* serum creatinine, *Ctnl* troponin, *CK* creatine kinase, *HBDH* hydroxybutyrate dehydrogenase, *LDH* lactate dehydrogenase, *PT* prothrombin time, *PTA* prothrombin time activity percentage, *INR* international normalized ratio

**Table 2:** Comparison of hematological parameters in survivals and non-survivals of STFS patients ($\overline{x}$ ±SD)

Parameters	Survival	Death	*p*
WBC (×10^9^/mL)	4.02 ± 2.71	4.14 ± 2.68	>0.05
NEU (%)	58.3 ± 17.1	68.5 ± 16.7	<0.01
LYM (%)	30.2 ± 13.4	21.6 ± 11.0	<0.01
N/L ratio	2.77 ± 2.50	4.74 ± 3.77	<0.01
MON (%)	10.78 ± 6.62	8.47 ± 6.36	<0.05
EOS (%)	0.08 ± 1.07	1.25 ± 1.73	>0.05
RBC (×10^12^/mL)	4.6 ± 0.53	4.39 ± 0.45	<0.05
Hb (g/L)	138.7 ± 18.1	138.5 ± 14.7	>0.05
HCT (%)	39.1 ± 4.5	38.6 ± 4.3	>0.05
MCV (fL)	85.0 ± 5.6	88.0 ± 6.4	<0.01
RDW (%)	13.3 ± 1.3	14.5 ± 7.9	<0.05
PLT (×10^9^/mL)	60.8 ± 35.6	40.2 ± 13.7	<0.01
PCT	0.07 ± 0.04	0.05 ± 0.03	<0.01
MPV (fL)	11.0 ± 1.0	10.8 ± 2.1	>0.05

*WBC* white blood cell, *NEU* neutrophil, *LYM* lymphocyte, *N/L ratio* neutrophil/lymphocyte ratio, *MON* monocyte, *EOS* eosnophils, *RBC* red blood cell, *Hb* hemoglobin, *HCT* hematocrit, *MCV* mean corpuscular volume, *RDW* red cell distribution width, *PLT* platelet count, *PCT* thrombocytocrit, *MPV* mean platelet volume

### Establishment of risk score model

Cox regression model analysis showed that baseline parameters, including age, serum AST level and sCr level were independent risk factors of mortality. HR (95% CI), and mean survival times of these factors are shown in Table 3. Age is the most remarkable risk factor among these parameters with HR = 1.128.

**Table 3:** Hazard ratio (HR) and means survival time of factors independently associated with mortality

Parameters	HR (95% CI)	Cutoff value	Mean time (days) (95%CI)	Log Rank	
				×^2^	*p*
Age (year)	1.128 (1.071,1.189)	≤66	28.8 ± 0.86 (27.2, 30.6)	28.3	<0.001
		>66	17.9 ± 1.72 (14.5, 21.2)		
AST (U/L)	1.002 (1.001,1.003)	≤437	28.6 ± 0.8 (27.0, 30.0)	55.1	<0.001
		>437	11.2 ± 1.76 (7.7, 14.6)		
sCr (μmmol/L)	1.013 (1.006,1.020)	<70	23.7 ± 0.66 (22.4, 25)	35.7	<0.001
		≥70	17.4 ± 1.96 (13.5, 21.2)		
M		≤10	30.3 ± 0.62 (29.1, 31.5)	88.4	<0.001
		>10	12.0 ± 1.83 (8.4, 15.6)		

The cutoff values and area under ROC curve (AUCs) of these parameters for predicting death are included in Table 4. AST level has the highest predictive value among these factors. Based on regression coefficients of multivariate Cox regression analysis of these factors, we constructed a risk score model for the prediction of mortality in SFTS patients.

**Table 4:** Cut-off values, AUCs of age and biochemical parameters for the prediction of mortality of SFTS patients with sensitivity and specificity

Parameters	cutoff value	AUCs (95%CI)	SEN (%)	SPE (%)	PPV (%)	NPV (%)	LR+	LR-
Age (year)	66	0.771 (0.701, 0.831)	75	70.2	42.9	90.4	2.51	0.36
AST (U/L)	437	0.797 (0.730, 0.854)	60	90.3	64.9	88.3	6.18	0.44
sCr (μmml/L)	70	0.764 (0.692, 0.827)	71.8	77.6	50.0	89.8	3.21	0.36
M	10	0.892 (0.829,0.955)	82.5	86.6	64.7	94.3	6.14	0.20

*AUC* area under, *ROC* curve, *SEN* sensitivity, *SPE* specificity, *PPV* positive predictive value, *NPV* negative predictive value, *LR+* positive likelihood ratio, *LR-* negative likelihood ratio

$$ \mathrm{Model}\left(\mathrm{M}\right) = 0.002 \times \mathrm{A}\mathrm{S}\mathrm{T} + 0.121 \times \mathrm{A}\mathrm{GE} \times + 0.013 \times \mathrm{s}\mathrm{C}\mathrm{r} $$

**Fig. 1:**
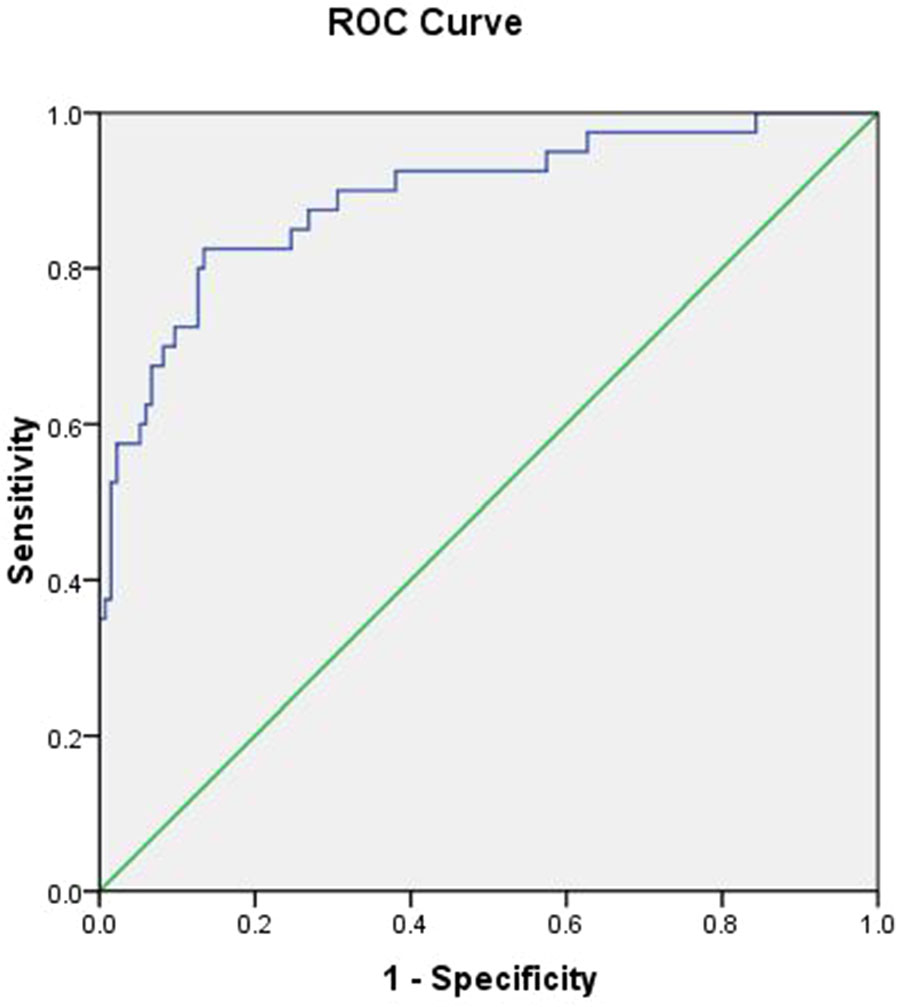
ROC curve and area under ROC curve of risk score model for the prediction of mortality of SFTS patients

**Fig. 2:**
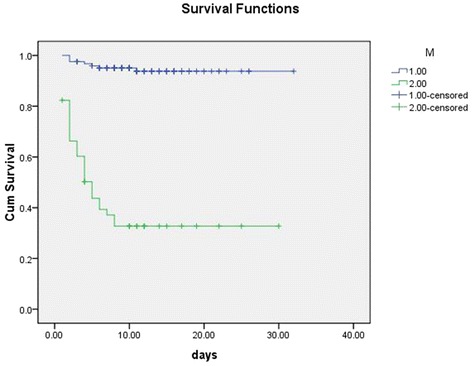
Survival curve of SFTS patients based on risk score model

The cutoff value of the score model was 10. Patients with >10 were at high-risk of mortality. AUC of the model for predicting mortality was 0.892, with sensitivity and specificity of 82.5 and 86.6%, respectively. Mean survival time of patients with M > 10 is shorter than those with M ≤ 10. Log-rank test indicated strong statistical significance (×^2^ = 88.35, *p* < 0.001). Data are shown in Tables 3 and 4. ROCs and survival curves are depicted in Figs. 1 and 2. Of those with M ≤ 10 (*n* = 123), 7 cases died (5.7%). And of those with M > 10 (*n* = 51), 33 cases died (64.7%). Mortality rates between two groups had strong significant difference (×^2^ = 70.9, *p* < 0.001).

### Prospective validation

The cutoff value of M (=10) was prospectively validated in a cohort of SFTS patients (*n* = 67) enrolled in our hospital this year. Of whom, 51 cases survived (76.1%) and 16 died (23.9%). Of those patients with M ≤ 10 (*n* = 39, 58.2%), 36 cases survived (92.4%) and 3 cases died (7.6%). Of those with M > 10 (*n* = 28, 41.8%), 13 cases died (46.4%) and 15 cases survived (53.6%). Mortality rates between these two groups had significant difference (×^2^ = 13.5, *p* < 0.001).

## Discussion

In this study, we established a risk model for the prediction of mortality of SFTS patients that showed relatively high predictive value with high sensitivity and specificity. The model contains 3 high-risk factors including age, serum AST level and sCr level, which have moderate predictive values for mortality when used independently. Numerous observational studies have shown that neurological symptoms were strongly associated with death. In this study, though not all the fatal patients have neurological symptoms at admission, all cases developed neurological symptoms afterwards. While, results showed that neurological symptoms was not an independent risk factor. The reason may be that neurological symptoms in survival patients were relative mild and the symptoms were severe in the death group. Ages of those critical patients with severe neurological symptoms who survived were younger than those who died. As we shown, age is the highest risk factor of the parameters. Elevated serum AST and sCr level may represent multi-organ dysfunction.

Other factors such as time intervals between symptom onset and treatment initiation, and therapeutic methods may also influence the prognosis of the patients. A delayed diagnosis of SFTS could affect the prognosis of the patients [4].

Other study showed that cytokines, such as interleukine (IL)-1, IL-6, IL-10, granulocyte colony stimulating factor (G-CSF), interferon inducible protein-10 (IP-10) and monocyte chemoattractant protein (MCP-1) were profoundly elevated in fatal cases, and their levels correlated with various clinical parameters [10]. While, the measurements of several serum cytokine levels together are too expensive to be performed by all healthcare facilities in China, so it is unsuitable to be used to predict the clinical prognosis.

Though only 58.6% patients had fever at adimissiom, and body temperature was not associated with prognosis, all patients had fever prior admission or body temperature elevated after admission. Fever and fatigue were the main chief complaint of SFTS patients. Though the fatality rate of SFTS in China has fallen from first reported 30% to present 12% owing to increased reporting, advances in diagnostic procedures and improved clinicians’ skills [5, 15, 16]. The risk factors for human infection with SFTSV were confirmed [15, 17] and national intervention programs were performed, including promoting public awareness, establishing sentinel hospitals and improving clinicians’ skills [2, 18–20]. Yet, the number of fatal cases increases annually in China.

Except for new treatment methods tried in a few therapies with few patients in Japan and South Korea [21–23], supportive care remains the standard treatment for SFTS. Therefore, accurate prediction of the prognosis may help clinicians to perform intervention measures in advance, control the disease progression and improve the prognosis.

In the prospective study, we showed that the model is more suitable for the prediction of survival than for mortality. The model we constructed will be helpful for the prediction of SFTS prognosis. And the results will be further prospectively validated by a more larger cohort of patients.

This study had several limitations. Firstly, we had no quantitative results of the virus RNA and only had the positive results. Secondly, patients with subclinical or minor infection signs who sought medical care were perhaps not tested for SFTSV. Thirdly, some patients with mild manifestations may have been excluded due to their low/undetectable viral loads. All these factors influence the accuracy of registered incidence and mortality.

## Conclusions

In summary, the risk score model that we established is useful for predicting death in patients with SFTS and may be applicable in clinical practice.

## Abbreviations

ALT: Alanine aminotransferase
AST: Aspartate aminotransferase
AUCs: Area under ROC curve
BUN: Blood urea nitrogen
CFR: Case fatality rate
CK: Creatine kinase
G-CSF: Granulocyte colony stimulating factor
HBDH: Hydroxybutyrate dehydrogenase
HR: Hazard ratios
hs-CRP: High-sensitivity C-reactive protein
IL-1: Interleukine-1
INR: International normalized ratio
IP-10: Interferon inducible protein-10
LDH: Lactate dehydrogenase
LYM: Lymphocyte
MCP-1: Monocyte chemoattractant protein
MCV: Mean corpuscular volume
N/L: Neutrophil/lymphocyte
NEU: Neutrophil
PLT: Platelet
PT: Prothrombin time
PTA: Prothrombin time activity
ROC: Receiver-operating characteristic
RT–PCR: Reverse transcription–polymerase chain reaction
sCr: Serum creatinine
SFTS: Severe fever with thrombocytopenia syndrome
TB: Total bilirubin
WBC: White blood cell
